# Audit cycle - VTE risk assessment in inpatient wards in mid Essex

**DOI:** 10.1192/bjo.2021.807

**Published:** 2021-06-18

**Authors:** Hesham Abdelkhalek

**Affiliations:** Essex Partnership University NHS Foundation Trust

## Abstract

**Aims:**

It is trust policy that the VTE risk assessment should be completed for every patient admitted to wards. The standard for this audit is therefore 100% completion. We completed the audit in October 2018 and closed the loop in September 2019.

**Method:**

This was a cross-sectional study of all patients on all the wards according to patients’ list on the electronic system (Paris) on certain date. In the first audit we used an audit tool from a similar audit performed in another area in the trust. For the purpose of re- audit we designed an audit tool to reflect the changes made in the electronic form.

**Result:**

In the re-audit, there was noticeable improvement in the completion rate compared to initial audit (95% vs. 82%); however, there was still under-performance. An interesting observation of the re-audit is that 74% percent of admissions had VTE risk assessments forms completed on same day of admission or next day compared to only 45% in previous audit.

**Conclusion:**

When looking at the completion of individual components on the VTE forms there are still some room for improvement as well. For example, in 26% of the patients there was no documentation about the use of prophylactic anticoagulants before admission compared to 34% in our previous audit. Also in 7% of the patients there was no documentation about the outcome of the assessment compared to only 3% in previous audit.

This is an audit to assess the completion of electronic VTE forms as per trust policy. Following the initial audit we made recommendations to improve completion rate. In the re-audit there was an improvement in total completion rate but we have not met the goal of 100% yet.

